# fMRI studies evaluating central respiratory control in humans

**DOI:** 10.3389/fncir.2022.982963

**Published:** 2022-09-23

**Authors:** Carolina Ciumas, Sylvain Rheims, Philippe Ryvlin

**Affiliations:** ^1^Department of Clinical Neurosciences, Lausanne University Hospital and University of Lausanne, Lausanne, Switzerland; ^2^Lyon Neuroscience Research Center, Institut National de la Santé et de la Recherche Médicale U1028/CNRS UMR 5292 Lyon 1 University, Bron, France; ^3^IDEE Epilepsy Institute, Lyon, France; ^4^Department of Functional Neurology and Epileptology, Hospices Civils de Lyon, Lyon, France

**Keywords:** central respiratory control, brainstem, fMRI, breathing, forebrain

## Abstract

A plethora of neural centers in the central nervous system control the fundamental respiratory pattern. This control is ensured by neurons that act as pacemakers, modulating activity through chemical control driven by changes in the O_2_/CO_2_ balance. Most of the respiratory neural centers are located in the brainstem, but difficult to localize on magnetic resonance imaging (MRI) due to their small size, lack of visually-detectable borders with neighboring areas, and significant physiological noise hampering detection of its activity with functional MRI (fMRI). Yet, several approaches make it possible to study the normal response to different abnormal stimuli or conditions such as CO_2_ inhalation, induced hypercapnia, volitional apnea, induced hypoxia etc. This review provides a comprehensive overview of the majority of available studies on central respiratory control in humans.

## Introduction

Regulating breathing is a response to alterations in blood levels of oxygen (O_2_) and carbon dioxide (CO_2_). Traditionally, this regulation process was attributed primarily on respiratory control centers located in the brainstem, particularly in the medulla and the pons. However, more recently, suprapontine structures such as the limbic areas, the diencephalon, the striatum and the cortex were ascribed to be essential in modulation of the respiratory drive of the brainstem (Horn and Waldrop, 1998; Pattinson et al., 2009b; Feldman et al., 2013). The basic pattern of respiration is generated in the medulla, and primarily regulated by pontine centers. These areas constantly regulate respiration, so that oxygen, carbon dioxide and acid levels are kept within normal limits. It is possible for someone to deliberately breathe faster or slower or to hold their breath, and it is also possible to not breathe at all for a period of time. This active control is regulated by the cerebral cortex, the amygdala and the hypothalamus, which participate in normal or exaggerated respiratory control, such as in stressful conditions (Horn and Waldrop, 1998). There are many brain regions sensitive to hypoxia and hypercapnia, and so the overriding of the will not to breathe comes from many regions (Guz, 1997). Both voluntary and automatic respiratory control systems are primarily integrated within the brainstem. However, in animal models, electrical stimulation of the cortex produces respiratory responses directly through the dorsal cord and the respiratory motor neurons and indirectly *via* the corticobulbar pathways (Shea, 1996). Brainstem reflex respiratory response is inhibited if cortical inputs are altered, as shown in decorticated animals (Tenney and Ou, 1977), in patients with bilateral infarction (Heyman et al., 1958) or in opioid administration (Pattinson et al., 2009a).

Several acute and chronic neurological conditions are associated with altered breathing patterns. This is caused by changes that occur in central respiratory control centers located in the brainstem or in the forebrain (Nogues and Benarroch, 2008). In general, these changes are less severe in chronic diseases, like multiple sclerosis, compared to acute diseases like stroke (Nogues et al., 2002). They can also occur intermittently, such as during or after an epileptic seizure, where they might lead to sudden unexpected death in epilepsy (SUDEP).

### Central nervous control

#### Medulla

**Figure 1:**
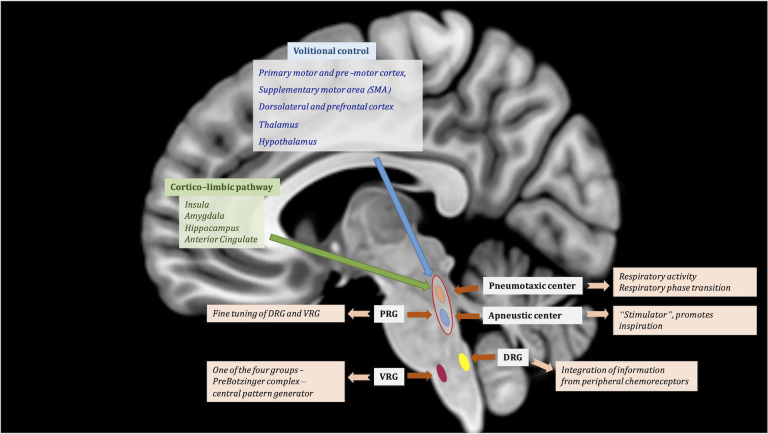
Central regulation of respiratory function. Suprapontine modulation of breathing through volitional control (blue) and corticolimbic pathway (green). Automatic regulation is assured by the brainstem centers contributing to the respiratory drive: (1) Pontine respiratory group (PRG) is represented by the pneumotaxic center and the apneustic center; and (2) Medulla—respiratory centers comprised of the dorsal respiratory group (DRG) and ventral respiratory group (VRG), the latter is part of the ventral respiratory column. The VRG is composed of four groups of neurons: (1) the Bötzinger complex; (2) caudal VRG; (3) the Prebötzinger complex; and (4) the rostral VRG. MNI T1 in sagittal projection was used for illustration.

Respiratory centers in the medulla are divided into the dorsal respiratory group (DRG) and the ventral respiratory column (VRC). The DRG represents the ventro-lateral portion of the nucleus tractus solitarius (NTS) and is mainly an inspiratory group (Alheid and Mccrimmon, 2008). It is described as a center of integration for afferents from peripheral chemoreceptors *via* the glossopharyngeal and vagus nerves (Alheid et al., 2011). The DRG sends constant bursts to respiratory motor neurons (Lalley, 1986). The VRC is a bilateral column formed by neurons in the lateral tegmentum and extending from the caudal part of the facial nucleus to the spino-medullary junction (Alheid and Mccrimmon, 2008; Figure 1). The caudal half of the VRC, termed the ventral respiratory group (VRG), contains bulbospinal respiratory premotoneurons that receive converging inputs from VRC rhythm generating neurons and from neurons outside the VRC, sculpting the activity pattern distributed to various pools of respiratory motoneurons (Alheid and Mccrimmon, 2008). The VRG is subdivided into the rostral (rVRG) and caudal (cVRG) group, based on the peak concentrations of inspiratory (rVRG) vs. expiratory (cVRG) bulbospinal neurons (Smith et al., 2013). The VRG is described as primarily expiratory, but it also contains inspiratory neurons. It consists of four groups of neurons, which generate a breathing rhythm through communication with each other: (1) the Bötzinger complex (BC); (2) caudal VRG jointly control the voluntary forced exhalation by sending input to intercostal and abdominal muscles. This is opposing: (3) the Prebötzinger complex (PBC); and (4) the rostral VRG that jointly acts to increase the force of inspiration (Ikeda et al., 2017). The PBC and BC are believed to be the central pattern generators of respiration (Smith et al., 2009).

#### Pons

The pons has two significant centers implicated in breathing regulation, both being part of the pontine respiratory group (PRG): (1) the pneumotaxic center; and (2) the apneustic center (Figure 1). The pneumotaxic center, located bilaterally in the dorsal rostral pons, is composed of the Kölliker-Fuse (KöF) and the parabrabrachial nuclei (PB) complex (Ikeda et al., 2017) and is involved in inspiratory off-switch. The KoF/PB complex is responsible for regulation of respiratory activity and respiratory phase transition (Ikeda et al., 2017). The apneustic center is located in the lower pons (Kahn and Wang, 1967). The PRG exerts “fine-tuning” influences over the medullary respiratory centers to help produce normal smooth inspirations and expirations (Douglas et al., 2004; Figure 1). Both centers communicate in order to control the rate and depth of breathing. The apneustic part is a “stimulator” and promotes inspiration by sending inputs to neurons in the DRG and VRG controlling inspiration. Although neurons involved with respiration are aggregated in certain parts of the brainstem, neurons that are active during inspiration are intermingled with those active during expiration (Smith et al., 2013).

### Chemical control

Of vital importance in the control of respiration are chemoreceptors. These receptors respond to the CO_2_ level in circulating blood, but the gas acts indirectly. CO_2_ is capable of diffusing through the capillary blood-brain barrier. In the blood, dissolved CO_2_ is neutralized by the bicarbonate-carbon dioxide buffer system and carbonic acid is formed, leading to the production of hydrogen and bicarbonate ions, and allowing the body to maintain a physiological pH (Alheid and Mccrimmon, 2008). When CO_2_ is elevated, the concentration of hydrogen ions in the blood increases, lowering pH and resulting in acidosis. The central chemoreceptors are stimulated and respond to this pH change. The rise in blood CO_2_ level, known as hypercapnia, thus triggers ventilation. The chemoreceptors that regulate respiration are located centrally near the medullary respiratory centers and peripherally in the arteries. Central chemoreceptors in the brainstem are continuously regulating breathing through monitoring of pH, the partial pressure of carbon dioxide (PCO_2_) and oxygen (pO_2_) in the blood. This regulation is insured by afferents from peripheral chemoreceptors located in the carotid body that are primarily targeting the solitary tractus (Lahiri et al., 1978). The partial pressure of oxygen (PO_2_) will stimulate respiration when it reaches severe hypoxemic levels (Javaheri and Kazemi, 1987). The medullar structures playing the role of central chemosensors responding to changes in pH and PaCO_2_ in arterial blood or cerebral spinal fluid (CSF) are: (1) the raphe nuclei; (2) the arcuate nucleus; (3) the retrotrapezoid nucleus (RTN); and (4) the parafacial respiratory group (pFRG; Smith et al., 2013). The raphe nuclei are partly formed of serotoninergic neurons that participate in cardiorespiratory regulation (Morris et al., 2010). The arcuate nucleus is a group of neurons involved in the breathing rate control and is located bilaterally on both sides of the midline in the medulla (Mikhail and Ahmed, 1975). The pFRG and RTN, the most rostral structures of the ventral medulla, are respiratory-modulators and are also regulated by hypercapnia (Smith et al., 2013).

Peripheral chemoreceptors that regulate breathing are found in structures known as the carotid and aortic bodies. These bodies contain sensory neurons which react primarily to a reduction in oxygen supply. They are generally not involved in regulating respiration, since they do not act until O_2_ drops to a very low level (Memmler et al., 1992). Recent studies have shown that astrocytes are also involved in chemosensing regulation in the brainstem. Low PO_2_ stimulates astrocytes in the brainstem, and they promote a general respiratory response to hypoxia to deliver adequate oxygen to arterial blood (Angelova et al., 2015; Sheikhbahaei et al., 2018). Since there is usually a sufficient supply of O_2_ in the blood, CO_2_ has the most immediate effect on regulating breathing in the central chemoreceptors. As the level of CO_2_ increases, increased respiration is required to remove excess gas. Chemoreceptors play an important role in developing the uncomfortable sensation of dyspnea, mostly due to direct connection to forebrain regions particularly in the limbic system, and they stimulate the respiratory system due to hypoxia/hypercapnia (Buchanan and Richerson, 2009).

### Cerebral and limbic system control

While it is universally accepted that the brainstem drives the autonomic respiratory pattern, cortical modulation of central respiratory rhythm and the conscious perception of breathing are still poorly understood (Evans, 2010). Through the cerebral cortex, it is possible to consciously or unconsciously increase or decrease the rate and depth of the respiratory movements. The extra-pontine and extra-bulbar respiratory centers are involved in *volitional control, autonomic control, and cortico-limbic control* (Figure 1).

The *cortico-limbic pathway* involved in modulation of respiratory control is very similar to the one active during strong emotional or affective states (Ledoux, 2000; Nagai et al., 2010; Feinstein et al., 2022). It is mostly represented by the insula and associated operculum (its anterior agranular part mostly), the head of the hippocampus, the amygdala, and the anterior cingulate cortex (ACC; Evans, 2010). Imaging studies have showed involvement of the amygdala in experimentally induced anxiety (Feinstein et al., 2022), of limbic structures in sleep disorders (Harper et al., 2014), and of the insula in obstructive sleep apnea (OSA; Li et al., 2015).

The *autonomic regulation* is mainly controlled by the brainstem, and it was mainly covered above, but inputs and outputs are generated by central structures acting in the control of cardio-respiratory functions, including the insula, temporal lobe, central operculum, and periaqueductal gray matter (Benarroch, 1993; Linnman et al., 2012). The fastigial nucleus of the cerebellum also contains CO_2_/H+ intrinsic chemoreceptors and plays an essential role in compensating for extreme changes in blood pressure and modulating hypercapnia-induced respiratory response *via* monosynaptic projections to the medullary gigantocellular nuclei (Martino et al., 2007). The cerebellum is also thought to participate mainly in the expiration phase of breathing (Prasad et al., 2021).

The *volitional respiratory control* is mediated by the primary motor and pre-motor cortex, the supplementary motor area (SMA), the dorsolateral and prefrontal cortex for decision making and motor planning, and by thalamic nuclei (ventro-posterolateral, ventrolateral, anterior, pulvinar), as well as the hypothalamus. Various studies highlight the essential role of the dorsomedial hypothalamus in respiratory regulation as a result of different types of stressful situations (for review see Dampney et al., 2008). The caudal hypothalamus is also responsible for integration of respiratory output in relation to changes in homeostasis (Horn and Waldrop, 1998).

Afferents from pontine and medullary respiratory centers, cortical, limbic and other suprapontine structures descend along the anterolateral column of the spinal cord to the phrenic, intercostal and abdominal muscle motor neurons and generate respiratory movements.

## Functional MRI of The Brainstem in Relation to Respiration

The original fMRI studies of respiration were designed to detect central respiratory control in humans, but suffered from very limited sample size, lack of statistical power, and difficult interpretation of clusters observed around the midline (Gozal et al., 1995; Harper et al., 1998; Evans et al., 1999; Šmejkal et al., 2000). Furthermore, there was no correction for the various artifacts (motion artifacts caused by breathing, cardiac movement, cerebral spinal fluid pulsation) that impact BOLD signals generated in the brainstem or suprapontine structures (Dagli et al., 1999).

In recent years, the quality of MR imaging of the midline structures has improved significantly through the development of high field and ultra-high field MRI. However, imaging of the human brainstem remains challenging. Very few structures or nuclei can be reliably identified on a structural MRI, even on a high-field MRI scanner, let alone on a 1.5 or 3T scan. This becomes even more challenging for fMRI studies, since the BOLD signal is far more difficult to detect in the brainstem than in cortical areas due to a much lower signal-to-noise ratio (Beissner et al., 2014). However, there are solutions to tackle these issues, such as reducing the size of the acquisition field of view, which will consequently increase the spatial resolution and lead to a smaller voxel size, or correcting images for physiological movements.

Spontaneous fluctuations of BOLD signal during fMRI acquisitions are influenced by a multitude of physiological variables, including cardiac rhythm (Piche et al., 2009), respiratory movements (Hu et al., 1995; Kruger and Glover, 2001; Birn et al., 2006), head movements and changes in CO_2_ (Iacovella and Hasson, 2011). The presence of major arteries and CSF in the vicinity of the brainstem adds to this physiological noise and further decreases the signal-to-noise ratio in fMRI studies of respiratory centers (Beissner et al., 2014; Beissner, 2015).

It is customary for fMRI studies dedicated to central respiratory control research to record all above-mentioned physiological signals to correct for their impact on BOLD signal. This requires using a pneumatic belt, end-tidal gas monitoring and head positioning foam pads during fMRI acquisition (Chang and Glover, 2009). De-noising of data from physiologically occurring signals can then be applied (Glover et al., 2000; Birn et al., 2006, 2008b; Chang and Glover, 2009). However, one needs to keep in mind that the removal of these signals, and in particular those related to respiratory movements, might partly hide the specific activation of respiratory centers elicited by the fMRI experiment (Iacovella and Hasson, 2011). Physiological noise can also be removed by computing both low frequency [physiological oscillations (~0.01–0.15 Hz)] and high-frequency (driven by cardiac rhythm and normal breathing) physiological regressors (Windischberger et al., 2002; Birn et al., 2008a, b; Chang and Glover, 2009; Chang et al., 2009; Yuan et al., 2013; Cordes et al., 2014). Removal of these components from the fMRI series can induce significant changes and allow for a more reliable interpretation of the BOLD signal (Chang et al., 2009; Tong et al., 2019). This is especially valid for the reduction of susceptibility artifacts occurring along the vertebrobasilar arterial system and neighboring brainstem (Dagli et al., 1999). There are also other susceptibility artifacts that can induce changes in the magnetic field, such as the oscillatory chest movement, diaphragm shifting, and changes in the inhaled/exhaled gas. These artifacts will generate respiration-induced *B*_0_ fluctuations and produce shifts in the phase of the MR image (Van De Moortele et al., 2002). A potential solution is parallel imaging, which increases the contrast to noise of echo-planar image (EPI) data by acquiring multiecho EPI (Poser et al., 2006). Multiecho EPI samples the data at multiple repeated short echo times (TE). This type of acquisition with optimized echo weighting can reduce susceptibility-induced distortion and dropout artifacts in EPI images, while improving BOLD contrast sensitivity (Poser et al., 2006). The technique, in combination with Independent Component Analysis (ICA), has been shown to be effective in significantly reducing susceptibility artifacts in the brainstem (Kundu et al., 2012; Beissner and Baudrexel, 2014).

While total removal of these artifacts is improbable, their reduction is feasible either during the acquisition or in the pre-processing stage, by applying different denoising algorithms (Caballero-Gaudes and Reynolds, 2017). Yet, the intrinsic temporal relationship between the activation of the brainstem respiratory centers and respiration related movements makes such denoising a risk of masking the BOLD signal of interest. In addition, one needs to consider the degrees of freedom lost in this process. One of the most commonly used method in the field is RETROICOR, which is based on the Fourier transformation of cardiac and breathing rhythmic activities to correct for their related movement artifacts (Glover et al., 2000). A modified RETROICOR method has been developed to avoid overfitting the noise from physiological signals (Harvey et al., 2008; Jones et al., 2008; Wallace et al., 2017). Other approaches use ICA, CompCor toolbox from the CONN toolbox, or masked ICA (mICA) with a brainstem mask, to perform a signal decomposition directly from the acquired images into various components, including physiological artifacts such as breathing or heartbeat (Beissner et al., 2014; Moher Alsady et al., 2016; Jarrahi, 2021). Overall, modified RETROICOR and mICA are thought to enable removal of respiration-induced movements without masking the activity of brainstem respiratory centers (Beissner et al., 2014).

Reducing physiological noise in the brainstem is necessary for studies investigating its functions, such as control of breathing (Dunckley et al., 2005; Harvey et al., 2008). Denoising is particularly important in distinguishing between the true response of respiratory centers and the spill-over effect from neighboring vessels (Khalili-Mahani et al., 2013). Only few structures within the brainstem can be reliably identified on the traditional MRI (Matt et al., 2019). Parcellation techniques, widely used for segmenting MRIs, usually have one single label for the brainstem, or in some atlases, the brainstem is segmented into midbrain, pons, and medulla. Comparison of different preprocessing techniques for the brainstem described five methods that were sensitive to brainstem activation, including use of various parameters for normalization and smoothing in several commonly used software packages (Beissner et al., 2011). The small size of brainstem respiratory centers represents a significant issue in fMRI studies of respiratory control. Indeed, classical gradient echo images have a low spatial resolution, 3 mm^3^ or lower (Weibull et al., 2008). This issue can be addressed by reducing the acquisition field of view by focusing on the brainstem (Pattinson et al., 2009b), or by using ultra-high field MR scanners which have provided promising results in the identification of brainstem nuclei. In addition, smoothing enables improving the high signal-to-noise ratio (SNR) of brainstem fMRI (Worsley and Friston, 1995; Beissner, 2015). Also, recent studies indicate that due to the particularly small size of nuclei involved in autonomic control, a small smoothing kernel of 6 mm for smoothing the data during preprocessing is advisable (Mckay et al., 2003, 2008; Evans et al., 2009; Hess et al., 2013). Yet, this results in blurring the distinction of closely spaced brainstem nuclei.

Correction models for respiratory rate and cardiac rhythm, along with direct measurements of CO_2_ have also been applied successfully to quantify cerebrovascular reactivity (CVR) in the cerebrum (Golestani et al., 2015; Moreton et al., 2016; Prokopiou et al., 2019; Golestani and Chen, 2020). CVR is defined as the percentage signal change in CBF per mmHg change in arterial partial pressure of CO_2_ (PaCO_2_; Poublanc et al., 2015). It is often referred to as the ability of cerebral blood vessels to undergo diastolic contraction under the influence of hypercapnic challenges (Liu et al., 2019). CVR is also accompanied by mild hypoxia (Tancredi and Hoge, 2013; Chan et al., 2020). CVR is a marker of vascular reserve, and is complementary to basal cerebral hemodynamic measurements such as cerebral blood flow (CBF) and cerebral blood volume (CBV; Liu et al., 2019). Hypercapnia induced by CO_2_ gas blend administration in patients with various neurological diseases effectively triggers CVR, enabling to evaluate their perfusion reserve (Spano et al., 2013). Cerebral regions that exhibit less or little BOLD CVR are usually affected in conditions such as gliomas, trauma, amyloid deposition, occlusion of major arteries etc. (Vernieri et al., 1999; Pindzola et al., 2001; Hsu et al., 2004; Ziyeh et al., 2005; Mandell et al., 2008). The CVR response to breathing challenges is different from the one induced by CO_2_ inhalation, suggesting that CVR measurements in pathologies affecting the respiratory system may be inaccurate (Ogoh et al., 2019). One important issue in fMRI studies of respiratory centers is to distinguish BOLD signal changes due to CVR from those reflecting activation of brain respiratory response. In task fMRI the increase in the cerebral metabolic rate of O_2_ and CBV is associated with increased local neuronal activity, which results in local decrease in the concentration of deoxyhemoglobin, which decreases the tissue-blood susceptibility differential. As a result there is a decrease in spin dephasing, and consequential BOLD signal increase (Bandettini et al., 1992). This process makes it feasible to measure the task related response from the brain areas involved in the processing of the task. For the CVR measurement, use of vasodilatory stimulus such as breath holding (BH) or exogenous CO_2_ gas will induce only slight changes to cerebral metabolic rate of O_2_ but a very robust global increased BOLD signal, primarily reflecting the augmentation of CBF (due to vasodilatation effect of the CO_2_). The distinction between these two is the local and global response to the task and the ways to measure the signal. The CVR BOLD quantifies the overall signal variance, the ratio between changes in the BOLD signal and end-tidal CO_2_ change, whereas the BOLD response of the respiratory centers measures the local changes in BOLD intensity, which has a typical temporal profile known as the hemodynamic response function (HRF).

Removal of global signal changes is also fairly common (Macey P. M. et al., 2004). For the correction of CO_2_ fluctuations on the fMRI signal, the HRF CO_2_ can be convolved with end-tidal CO_2_ data, and the output regressed out of the BOLD signal (Prokopiou et al., 2019). Use of end-tidal CO_2_ measurements can also be useful when the user requires creation of the estimated CO_2_ arrival time at each brain region and quantification of the hemodynamic response following elevation of CO_2_ (Yao et al., 2021).

Using fMRI, there are several ways to obtain a response from the respiratory centers of the brain. This can be done either by: (i) inducing hypercapnia (through inhaling high concentration of CO_2_ or through imposing prolonged breath holding); (ii) inducing hypoxia (through reducing the concentration of inhaled O_2_); or (iii) through voluntary modulations of breathing (i.e., hyperpnea, breath-holding, slowing of respiratory pace). In particular, compliance with the breath-holding task is critical, given the role of prolonged volitional control in triggering an effective BOLD response from the respiratory centers. Breath-holding experiments can be either timed, using a fixed BH duration, or maintained for as long as participants can feel comfortable (Thomason et al., 2005; Mckay et al., 2008). Other methods to activate brainstem respiratory centers include hypercapnia and hypoxia challenges. Hypercapnia can be induced by administering a mixed gas with high concentration of CO_2_ (5%) and 95% O_2_, delivered for 2 min (Harper et al., 2005). For the hypoxia challenges, several paradigms have been used, including breathing a mixed concentration of 15% O_2_ and 85% N_2_ for 2 min (Macey et al., 2005), or undergoing five hypoxic episodes of breathing a mixed gas with 10% O_2_ and 90% N_2_ for 180 s followed by 90 s of normoxia (Gerlach et al., 2021). Because task-related changes in arterial gases, and notably pCO_2_, can be affected by manipulation of breathing, some respiratory imaging studies have used mechanical ventilation as a passive condition and volitional control of respiration as an active condition, which allowed observing BOLD signal activations, unaffected by manipulation of pCO_2_ (Ramsay et al., 1993; Evans et al., 1999).

Some of these studies are reviewed below.

## Hypercapnia

Increased levels of pCO_2_ and reduced pH trigger a response from the raphe pallidus, pFRG/RTN, and the NTS (Okada et al., 2008). Stimulation of these medullary chemoreceptors through an increase in pCO_2_ leads to an excitatory response from the respiratory neuronal network, and to a hypercapnic ventilatory response (*in vivo* and *in vitro* study; Gourine et al., 2005; Fukushi et al., 2021). Usually, elevation of pCO_2_ induces dyspnea or breathlessness in healthy subjects (Chonan et al., 1987; Burki and Lee, 2010) and this is used as a stimulus in investigations directed toward studying central respiratory control in dyspnea or air hunger (behavioral and pharmacological studies). The regional fMRI signal responses to hypercapnia, using 5% CO_2_-95% O_2_ for 120 s mixture, showed a pronounced increased response in regions not classically associated with breathing control, but traditionally related to affect, autonomic regulation, or motor coordination (Harper et al., 2005; i.e., the thalamus, dorsal striatum, insula, hippocampus, cingulate cortex, amygdala and hypothalamus; Brannan et al., 2001; Liotti et al., 2001; Von Leupoldt and Dahme, 2005). Limbic structures are also involved in breathing regulation such as air hunger (Banzett et al., 2000; Brannan et al., 2001; Liotti et al., 2001), urge-to-cough (Mazzone et al., 2007), or respiratory challenges such as induced hypercapnia by inhaling 5% CO_2_ (Harper et al., 2005) or inspiratory breath holding (Macefield et al., 2006) and forced expiratory loading (Macey K. E. et al., 2004). Limbic activations are more notable in highly anxious subjects compared to low anxious participants when subjected to hypercapnia (Chan et al., 2019). The cerebellar and more rostral (midbrain, pons) involvement in mediating hypercapnia was also reported (Gozal et al., 1994; Harper et al., 1998, 2005; Kastrup et al., 1999b; Brannan et al., 2001; Parsons et al., 2001).

Hypercapnia-based studies are typically confronted by questions on how to obtain the response—either by volitional manipulation—end-expiratory or end-inspiratory BH, hyperventilation or by CO_2_ gas mixture (which requires special equipment for the gas delivery, calibration, MR-safety measurements of all equipment etc). The advantage of such delivery is the control of inhaled and exhaled gas (Moreton et al., 2016), whereas the main constraint is the requirement of special equipment. Voluntary manipulations of breathing rely fully on subjects’ compliance, which also can be viewed as a constraint. The advantage is that it can be easily implemented by means of finding a suitable stimulus onset/offset software and deciding upon which type of breathing manipulation to use in the experimental setting. Studies cited above have studied CVR (Okada et al., 2008) or central response to hypercapnia (Harper et al., 2005), voluntary expiration (Macey K. E. et al., 2004), inspiratory occlusion (Chan et al., 2019), Valsava maneuver (Harper et al., 1998), end-expiratory BH (Kastrup et al., 1999b). Few studies were conducted using CO_2_ gas mixture (Gozal et al., 1994; Harper et al., 1998, 2005; Brannan et al., 2001; Liotti et al., 2001; Parsons et al., 2001). Some reference articles were using a PET technique (Brannan et al., 2001; Liotti et al., 2001; Parsons et al., 2001). Regarding the preprocessing of the data, some authors used data motion correction and global signal change removal (Macey K. E. et al., 2004; Harper et al., 2005), while some opted for no correction (Harper et al., 1998; Kastrup et al., 1999b; Chan et al., 2019), or only the motion correction (Gozal et al., 1994).

## Hypoxia

Hypoxia, reduction in pO_2_, is detected by the carotid chemoreceptors, which trigger a response from the brainstem respiratory centers that leads to hyperventilation, for review please see Gourine and Funk (2017). Another review points to peripheral chemoreceptors, that also trigger forebrain response, descending through the hypothalamus to the DRG and VRG (Fukushi et al., 2021). Indeed, the hypothalamus is one of the key regions driving the central respiratory response to hypoxia (Horn and Waldrop, 1998). Hypoxia is an effective way to experimentally trigger dyspnea, and can be easily reversed with inhalation of O_2_ (Fukushi et al., 2021). In an fMRI study, hypoxia was induced by repeated inhalation of 10% oxygen and 90% nitrogen, and contrasted with normoxia (Gerlach et al., 2021). Five distinct hypoxia-responsive regions were detected around the NTS (the nucleus ambiguous, intermediate reticular nucleus, dorsal motor nucleus of the vagal nerve, spinal trigeminal nucleus, and the inferior olivary nucleus; Gerlach et al., 2021), as well as three hypothalamic regions (the arcuate nucleus, anterior hypothalamic area/lateral hypothalamic area, and paraventricular nucleus; Gerlach et al., 2021). However, they did not monitor the end-tidal pCO_2_ during their experiment, nor did they apply a physiological correction to the BOLD series, raising the possibility that the central chemoreflex response could have been merged with the activations detected during their experiment. They also employed mICA focusing on the lower brainstem and the hypothalamus. As for the hypercapnia induced by CO_2_ inhalation, the advantage of inducing the hypoxia with a mixture of 10% O_2_ and 90% N_2_ is that it allows full control over the inhaled gas. The issue is that this method of gas delivery requires special MR safe equipment.

## Voluntary Modulation

Forebrain regions, such as the primary motor cortex, premotor area and supplementary motor area, are activated by volitional breathing (Brannan et al., 2001; Liotti et al., 2001; Von Leupoldt and Dahme, 2005). Voluntary modulation of breathing in 20 healthy volunteers who breathed at a slower pace than usual (i.e., 5.5 breaths per minute, similar to yoga practice, compared to 10 breaths per minute) resulted in increased BOLD activation pattern within the brainstem, across the dorsal length of the pons, in hypothalamic and thalamic regions, within cerebellar vermis and lateral cortices and in the striatum, the hippocampus and the motor, supplementary motor and parietal cortices (Critchley et al., 2015). These areas are part of an executive homeostatic network (Zaccaro et al., 2018). The observed activations hint to the link between the control of breathing and the baroreflex sensitivity. Hypoxic challenge (breathing 13% of O_2_) in the same group of subjects led to activation within the dorsal pons, bilateral amygdala, thalamus and cerebellar cortices, along with activation of occipital, medial and dorsolateral prefrontal regions, an activation pattern typically observed in stressful conditions (Critchley et al., 2015). In the modeling of fMRI data, they added end-tidal CO_2_, arterial oxygen saturation SaO_2_, respiratory rate, tidal and minute volume (ventilation), heart rate and standard deviation of inter-beat interval. Another modulation of breathing, which consists of 6 s breath (3 s breathe in and 3 s breathe out) or 12 s breath (6 s breathe in and 6 s breathe out), provided comparable end-tidal CO_2_ values and patterns of BOLD response compared to those resulting from inhalation of CO_2_ (Liu et al., 2020). Authors accounted for variation of end-tidal CO_2_ in their data preprocessing. An early fMRI study performed in five healthy men assessing volitional inspiratory control (voluntary hyperpnea ensured by a ventilator with creation of large tidal inspiratory volumes compared to passive expirations) showed activation within the superior motor cortex, premotor cortex and supplementary motor area (Evans et al., 1999). They used a 15 mm smoothing kernel, and no correction for any susceptibility artifacts was applied, but also no activation in the brainstem was reported. In another study where healthy participants executed voluntary hyperpnea (about three times faster as the normal breathing), the same cortical areas, along with medullar activation were detected (Mckay et al., 2003). They accounted for global signal changes in the volumes, and used a much smaller smoothing kernel—6 mm. Another paradigm consisted in contrasting unconscious to conscious breathing, by asking healthy volunteers to focus their attention (or not) on each inspiratory and expiratory movements. This resulted in modulation of the activation pattern in the premotor and parietal cortex (Šmejkal et al., 1999, 2000). Authors did not provide any information about data preprocessing.

The advantage of using voluntary respiration modulation relies on: (1) very little equipment required to perform the task; and (2) the facility to train subjects to perform the experiment. The drawback of this approach is: (1) little control over compliance; and (2) variability in subjects’ ability to hold long breath holds.

## Resting State fMRI

Resting state fMRI enables study of the synchronous spontaneous fluctuations between various cortical regions (Biswal et al., 1995). Several resting state networks have been described, however most studies focus on the default brain mode (DMN; Greicius et al., 2003). In the DMN, consistent regions of the brain are active at rest but reduce their activity when cognitive tasks are carried out. Abnormal resting state connectivity has been observed in various diseases, including conditions where respiratory and cardiovascular regulation is impaired. Confounds, such as bulk motion, cardiac-related motion, white matter fluctuations, respiratory-related motion, and variations in end-tidal CO_2_ account for 46% of signal variance in resting state fMRI data and must be considered when investigating low frequency variations of the BOLD signal (Harita and Stroman, 2017). Modulation of respiration also proved to influence resting state fMRI, with enhanced connections when pCO_2_ is increased (Mcketton et al., 2021). Authors corrected for the end-tidal PCO_2_ and for head motion, and used CompCor toolbox to extract physiologically related noise in the data. The same correction was used in a recent study, which reported that voluntary normal breathing through the mouth compared to nose breathing also resulted in increased functional connectivity (FC) throughout the DMN nodes (Jung et al., 2020). The authors linked this finding to the potential cognitive disturbances observed in subjects suffering from mouth breathing syndrome (Jung et al., 2020). Another study showed synchronized neural activity through a distributed network of limbic/paralimbic and brainstem regions during uninterrupted spontaneous respiration (Evans et al., 2009). Authors used a 6 mm kernel for smoothing, and used global regressors for correcting for variation of BOLD globally and the PCO_2_ regressor.

## Breath-Holding fMRI

Breath holding (BH) is commonly used to simulate the effect of apnea and hypercapnia on the brain, resulting in autonomic down-regulation of heart rate, vasodilatation and simultaneous reduction in blood flow to the brain (Kastrup et al., 1998; Corfield et al., 2001). Breath-holding results not only in rapid increase in pCO_2_ but also in reduction of PO_2_ in the first 20–30 s (Dubois, 1952; Hong et al., 1971), and the cumulative amount of CO_2_ for longer BHs (30–40 s) is lower than when the BHs last 20 s or less (Lindholm and Linnarsson, 2002). Hyperventilation before BHs will reduce the CO_2_ and increase the reserve of O_2_, and will diminish the urge to breath. While the PO_2_ levels after very long BHs will descend to 20 mmHg, the levels of CO_2_ will stay normal or slightly decreased (Lindholm and Lundgren, 2006). The breaking point of BH happens when the sum of lower PO_2_ and higher CO_2_ is sufficient to induce alveolar ventilation eight times higher the normal (Otis et al., 1948). The increase in PCO_2_ will stimulate the central respiratory rhythm, which keeps its rhythmicity throughout the BH and cannot be controlled voluntarily (Parkes, 2006). The breathing patterns correlate with fluctuations in PO_2_ and PCO_2_ during BH, which are vasoactive triggers that modify global cerebral perfusion (Kastrup et al., 1999c). While the majority of fMRI studies cited in this review had reported PCO_2_ or end-tidal CO_2_ variations measured in their studies, only very few also focused on PO_2_ variations (Gozal et al., 1995; Chan et al., 2020). That is probably due to the common concept that an increase in PCO_2_ will imminently decrease the PO_2_, as they work synergistically in the normoxic condition to stimulate peripheral chemoreceptors (Lahiri et al., 1978). Comparison of studies with and without measurement of PO_2,_ but with measurement of PCO_2_ showed that variation of PCO_2_ during BH and after BH are very similar (Otis et al., 1948; Gozal et al., 1995; Chan et al., 2020). Studies in which PO_2_ was measured (Gozal et al., 1995; Chan et al., 2020) indicated that hypoxia and hypercapnia have synergistic effect, and reproduce results observed in animal studies (Honda et al., 1963; Lahiri et al., 1978; Chan et al., 2020).

Compared to inhalation of CO_2_, where special equipment is needed, BH is safe, easy to use, suitable for various age groups, and offers fairly robust results (Godfrey and Campbell, 1968; Strohl and Altose, 1984; Li et al., 1999; Liu et al., 2002; Parkes, 2006; Mckay et al., 2008; Magon et al., 2009; Roberts et al., 2009; Bright and Murphy, 2013; Sutterlin et al., 2013; Tancredi and Hoge, 2013; Iranmahboob et al., 2016). Strong cortical, subcortical and medullary activations are usually observed during BH (data corrected for global intensity and smoothed with 6 mm kernel; Mckay et al., 2008) and the signal increase occurs with no apparent change in mean arterial pressure, but no information about data correction was reported (Kannurpatti et al., 2002). BH can be performed in different ways, either by: (1) end-expiratory BH (shortest due to lack of inhibitory lung stretch and a small lung volume reservoir to mix atmospheric air with arterial blood); (2) end-inspiratory BH (longer than expiratory BH); (3) end-inspiratory BH with previous hyperventilation (results in reduction of CO_2_ due to hyperventilation, which allows this type of BH to last longer); and (4) end-inspiratory BH with previous hyperoxia [similar to (3)] (Skow et al., 2015). The most commonly used BHs in fMRI are standard end-expiratory and end-inspiratory BHs. Computer paced end-expiratory BHs were suggested to show a more intense BOLD signal compared to self-paced breathing, and data were corrected for the delay in onset of the BH (Scouten and Schwarzbauer, 2008). Breathing pace in inspiratory BH also influences the level of fMRI activation, with greater BOLD changes with faster breathing when data was corrected for head motion and delay in BH onset (Chen et al., 2021). A recent review recommended to practice expiratory BHs of 15 s with self-paced recovery period for optimal results (Pinto et al., 2021). BH fMRI studies can be applied in younger populations but suffer from significantly noisier and less activated voxels in children than in adults (Thomason et al., 2005).

A very early fMRI study (1993) of the effect of hypoxia following inspiratory BH reported a decrease in the intensity of BOLD, but no information about correction applied is provided in the article (Stehling et al., 1993). However, subsequent fMRI studies that used the inspiratory and expiratory BH maneuvers consistently observed an increase in activations, however no particular correction for the physiological noise or else was used (Kastrup et al., 1998, 1999a; Li et al., 1999). Yet, end-expiratory BH was found to be associated with both increased BOLD response in the right insula, dorsal anterior cingulate, cerebellum, and fronto-parietal cortex, and decreased BOLD signal in the left insula, ventral anterior cingulate, precentral gyrus and hippocampus (Kimmerly et al., 2013; Sharman et al., 2014). Reported data was corrected for global signal intensity change (Kimmerly et al., 2013) and for respiration and heartbeat (Sharman et al., 2014). These regions are viewed as an autonomic cortical network controlling apnea-induced muscle sympathetic nerve activity (Kimmerly et al., 2013). Inspiratory BH was associated with fMRI activations in the midbrain, pons, cerebellum and lentiform nuclei, but no correction for the data was reported (Gozal et al., 1995). During resistive inspiratory load, higher BOLD activations were observed in ventrolateral and dorsal medulla (PBC) than in caudal ventro-lateral pons (pFRG; Hess et al., 2013). Data were corrected for respiratory volume per time, end-tidal CO_2_, RR cardiac interval and saturation, and a small smoothing kernel was used, 6 mm.

Most of the studies examined here took into account the variation of end-tidal CO_2_, likely because it is easy to measure and reflects the variation of O_2_ in expired air. However, as discussed at the beginning of this section, CO_2_ and O_2_ fluctuations will also affect the BOLD signal and these confounds should be considered as part of the data model (Moreton et al., 2016). The first fMRI studies of central control of breathing have used little or no correction for susceptibility and movement artifacts, and also suffered from poor spatial resolution. Due to these limitations, findings from these pioneering studies were difficult to interpret, both in terms of the mechanisms underlying changes in BOLD signal and their precise anatomical location. Yet, most of these findings were confirmed in subsequent studies which have applied an appropriate methodology (Sharman et al., 2014; Chen et al., 2021).

## fMRI of Central Respiratory Control in Different Diseases

### Sudden unexpected death in epilepsy (SUDEP)

Sudden unexpected death in epilepsy (SUDEP) is the most shattering outcome in patients with epilepsy, typically affecting adolescents and young adults between 20 and 40 years of age with drug resistant epilepsy. SUDEP may account for up to 1/3 of all causes of non-suicidal, non-accidental sudden death in this age range. Recent progresses have pointed to the primary role of post-ictal central respiratory distress (Patodia et al., 2021). Individuals who are at high risk of SUDEP, based on various clinical risk factors, exhibit regional brain structural and FC alterations compared with low-risk patients. In the former, FC was found reduced between pons and thalamus, between midbrain and thalamus (Tang et al., 2014), between the thalamus, brainstem, anterior cingulate, putamen and amygdala, and was elevated between medial/orbital frontal cortex, insula, hippocampus, amygdala, subcallosal cortex, brain stem, thalamus, caudate, and putamen (Allen et al., 2017, 2019). Structural alterations were also identified in patients who died from SUDEP, such as the presence of brainstem atrophy (Mueller et al., 2014) and reduced posterior thalamic gray matter volume, possibly linked to hypoxic challenges from apnea (Wandschneider et al., 2015). Finally, in a mixed population of SUDEP cases and individuals at high risk of SUDEP, fMRI showed impaired communication between several nodes involved in respiratory and cardiovascular regulation (La et al., 2019).

## Congenital Central Hypoventilation Syndrome

Congenital central hypoventilation syndrome (CCHS) is a genetic condition characterized specifically by a lack of sensitivity to CO_2_ and is defined by an important alteration of the automatic control of breathing. Hypoxia, induced through inhalation of 10% oxygen and 90% nitrogen, elicited comparable BOLD responses in CCHS and control subjects in medullary and hypothalamic structures but significantly different patterns in cerebellar, dorsolateral pontine, thalamic, basal ganglia, limbic, and midbrain areas (Macey et al., 2005) and the prefrontal cortex (Zhang et al., 2011). The altered suprapontine control of respiratory function could also account for a dysfunction in the other cognitive processes supported by the same brain regions. In a single CCHS case study, spontaneous breathing showed an increased functional connectivity between the brainstem and the frontal cortex, whereas assisted breathing (mechanical ventilation) resulted in restoration of physiological DMN low frequency oscillations and improved patients’ executive functions (Sharman et al., 2014). During Valsalva maneuvre, a forced exhale and hold task, the overall BOLD response in nine patients with CCHS was muted compared to healthy controls, leading authors to conclude that in CCHS the structures that mediate sympathetic and parasympathetic output are impaired (Ogren et al., 2010).

## Obstructive Sleep Apnea

Obstructive sleep apnea (OSA) is characterized by episodes of complete or partial repetitive upper airway collapse during sleep. In 12 patients with OSA, the BH maneuver resulted in less CVR than in controls, while brain regions of decreased CVR were larger in these patients than in controls (Buterbaugh et al., 2015). Expiratory loading in nine patients with OSA resulted in decreased global gray matter signal intensity, which was less pronounced than in healthy participants, and occurred twice as fast in OSA than in controls (20 s into the challenge compared to 40 s in controls; Macey et al., 2003). During this expiratory loading challenge, patients with OSA also showed increased activation in the ventral midbrain and the hippocampus, and decreased activation in the middle frontal gyrus and Broca’s area, insula and anterior cingulate (Macey et al., 2003). Inspiratory loading in seven patients resulted in decreased BOLD signal in the dorsal and ventral striatum, frontal cortex, insula, hippocampus and midbrain, while there were significant increases in activation in the dorsal midbrain, medial cingulate, temporal and cerebellar cortex (Macey et al., 2006). fMRI study using the Valsava maneuver in 21 male subjects with OSA showed a decreased response compared to controls in the left inferior parietal cortex, anterior superior temporal gyrus, posterior insular cortex, cerebellar cortex, fastigial nucleus, and hippocampus (Henderson et al., 2003). Also, in patients with OSA, the DMN pattern is selectively altered (Li et al., 2016; Wu et al., 2020). The areas that show altered BOLD response are usually expressing regional structural changes in the white and gray matter and reduced cortical thickness (Macey et al., 2008, 2018; Canessa et al., 2011).

## Conclusion

MR imaging in humans has demonstrated its ability to investigate the neural centers involved in the CNS control of respiration, using various types of experimental protocols. In particular, controlled hypercapnia, hypoxia, as well as breath holding maneuvers enable fMRI activation of brainstem respiratory centers and their hypothalamic, limbic and cortical controlling networks. Such investigations can now be used to explore medical conditions associated with known or suspected dysfunction of central respiratory control, in order to better understand their pathophysiology and to develop novel clinically relevant biomarkers.

## Author Contributions

CC collected books and articles for the review, conceived, designed, and wrote the review. SR and PR provided a methodological/clinical perspective and editing of the review, provided general advice on the review. All authors contributed to the article and approved the submitted version.

## Funding

The study was supported by two grants from InnoCentive Challenges—The SUDEP Institute Challenge 9933784: Developing Predictive Biomarkers of Epilepsy Seizures. Open access funding was provided by the University of Lausanne.
