# Erratum: fMRI studies evaluating central respiratory control in humans

**DOI:** 10.3389/fncir.2023.1176605

**Published:** 2023-03-13

**Authors:** Frontiers Production Office

**Affiliations:** Frontiers Media SA, Lausanne, Switzerland

**Keywords:** central respiratory control, brainstem, fMRI, breathing, forebrain

An omission to the funding section of the original article was made in error. The following sentence has been added: “Open access funding was provided by the University of Lausanne.”

The original article has been updated.

